# Apoptosis inhibitor-5 overexpression is associated with tumor progression and poor prognosis in patients with cervical cancer

**DOI:** 10.1186/1471-2407-14-545

**Published:** 2014-07-28

**Authors:** Hanbyoul Cho, Joon-Yong Chung, Kwon-Ho Song, Kyung Hee Noh, Bo Wook Kim, Eun Joo Chung, Kris Ylaya, Jin Hee Kim, Tae Woo Kim, Stephen M Hewitt, Jae-Hoon Kim

**Affiliations:** Department of Obstetrics and Gynecology, Gangnam Severance Hospital, Yonsei University College of Medicine, 146-92 Dogok-Dong, Gangnam-Gu, Seoul 135-720 South Korea; Tissue Array Research Program, Laboratory of Pathology, Center for Cancer Research, National Cancer Institute, National Institute of Health, Bethesda, MD 20892 USA; Department of Biomedical Sciences, Graduate School of Medicine, Korea University, Seoul, Korea; Department of Biochemistry, Korea University College of Medicine, Seoul, Korea; Radiation Oncology Branch, Center for Cancer Research, National Cancer Institute, National Institute of Health, Bethesda, MD USA

**Keywords:** API5, pERK1/2, Prognosis, Cervical cancer, Tissue microarray, Immunohistochemistry

## Abstract

**Background:**

The apoptosis inhibitor-5 (API5), anti-apoptosis protein, is considered a key molecule in the tumor progression and malignant phenotype of tumor cells. Here, we investigated API5 expression in cervical cancer, its clinical significance, and its relationship with phosphorylated extracellular signal-regulated kinase 1 and 2 (pERK1/2) in development and progression of cervical cancer.

**Methods:**

API5 effects on cell growth were assessed in cervical cancer cell lines. API5 and pERK1/2 immunohistochemical staining were performed on a cervical cancer tissue microarray consisting of 173 primary cervical cancers, 306 cervical intraepithelial neoplasias (CINs), and 429 matched normal tissues.

**Results:**

API5 overexpression promoted cell proliferation and colony formation in CaSki cells, whereas API5 knockdown inhibited the both properties in HeLa cells. Immunohistochemical staining showed that API5 expression increased during the normal to tumor transition of cervical carcinoma (*P* < 0.001), and this increased expression was significantly associated with tumor stage (*P* = 0.004), tumor grade (*P* < 0.001), and chemo-radiation response (*P* = 0.004). API5 expression levels were positively associated with pERK1/2 in cervical cancer (*P* < 0.001) and high grade CIN (*P* = 0.031). In multivariate analysis, API5+ (*P* = 0.039) and combined API5+/pERK1/2+ (*P* = 0.032) were independent prognostic factors for overall survival.

**Conclusions:**

API5 expression is associated with pERK1/2 in a subset of cervical cancer patients and its expression predicts poor overall survival, supporting that API5 may be a promising novel target for therapeutic interventions.

**Electronic supplementary material:**

The online version of this article (doi:10.1186/1471-2407-14-545) contains supplementary material, which is available to authorized users.

## Background

Cervical cancer is the second most common cancer in women worldwide [1]. Optimal treatment of early-stage cervical cancer is either radical surgery or radiotherapy. However, effective treatment options for patients with locally advanced cervical cancer are limited [2]. As 99% of cervical lesions contain viral sequences, the causative agent of cervical cancer is considered a persistent infection with high-risk subtypes of human papillomavirus (HPV) [3]. HPV oncoproteins E5, E6 and E7 are the primary viral factors attributed to the immortalization and malignant transformation of cervical cells. They present growth-stimulating and transforming properties [4]. HPV oncogene subtype, E6 and E7, interfere with cellular functions of tumor suppressor proteins and extend the proliferative capacity of infected cells by blocking apoptosis [5].

Apoptosis, also known as programmed cell death, plays a crucial role in development, morphogenesis, normal cell turnover and immune system function [6]. Acquired resistance to apoptosis is a well-known hallmark of cancer and contributes to tumorigenesis and the malignant phenotype [7]. Extracellular signal-regulated kinases 1 and 2 (ERK1/2) are members of mitogen activated protein kinase (MAPKs), mediates cell proliferation. The activation of ERK1/2 induces metaplasia and development of tumors via cell cycle arrest and apoptosis inhibition [8, 9].

Many oncogenes and tumor suppressor genes have been discovered and implicated in the regulation of apoptosis. Among these anti-apoptotic proteins, apoptosis inhibitor-5 (API5), also called anti-apoptosis clone 11 (AAC11), or fibroblast growth factor-2-interacting factor (FIF), is a nuclear protein. API5 expression has been shown to prevent apoptosis after growth factor deprivation [10]. The mechanism by which API5 prevents apoptosis is poorly understood, but Morris *et al*. recently showed that its anti-apoptotic action appears to be mediated by the negative regulation of transcription factor E2F1-induced apoptosis [11]. Furthermore, a recent study revealed that API5 contributes to E2F1 transcriptional activation of cell cycle-associated genes [12]. API5 has been reported to be up-regulated in multiple cancer cell lines, some metastatic tumor within lymph node tissues, and B cell chronic lymphoid leukemia [10, 11, 13–15]. However, there is no clear evidence showing API5 role in tumor progression of cervical cancer. Immune escape has been demonstrated as important in tumor progression especially in virus induced tumor such as cervical cancer. In this context, our recent study showed that API 5 acts as an immune escape gene by rendering tumor cells resistant to apoptosis triggered by tumor antigen-specific T cells. This effect was associated with pERK-dependent degradation of a pro-apoptotic molecule, BIM [16]. In this report, we aimed at investigating the clinical significance of API5 and its relationship with phosphorylated ERK1/2 (pERK1/2) in development and progression of cervical cancer.

## Methods

### Patients and tumor samples

In this study, 173 cervical cancer and 306 cervical intraepithelial neoplasia (CIN) cases were prospectively collected from patients who enrolled in Gangnam Severance Hospital, Yonsei University College of Medicine from March 1996 to March 2010, and received primary surgery during that time. All tumor tissues were histologically reviewed and only specimens with sufficient presence of tumor cells were included for tissue microarray (TMA) construction. Cervical cancer patients were clinically staged according to the International Federation of Gynecology and Obstetrics (FIGO) staging system. The treatment of cervical cancer consisted of radical hysterectomy with pelvic lymph node dissection via laparotomy for FIGO stage I/II. Adjuvant radiotherapy or platinum-based concurrent chemo-radiation was performed in cases with increased risk of recurrent disease, such as positive resection margins, positive lymph nodes, or parametrial involvement. Chemo-radiation therapy consisted of 40 mg/m^2^ cisplatin i.v. once a week for 6 weeks concomitantly with external pelvic and intracavitary radiation. For FIGO stage III/IV cervical cancer primary chemo-radiation therapy was generally recommended. Clinicopathologic factors including age, Hybrid Capture^®^ 2 (HC2) result, surgical procedure, chemo-radiation response, survival time, and survival status were obtained by reviewing medical records and pathology reports. Response to therapy was assessed according to Response Evaluation Criteria in Solid Tumors (RECIST; version 1.0), either by computed tomography or magnetic resonance imaging [17]. Chemo-radiation response was determined by locoregional recurrence with a follow-up time of at least 2 years. Tissue samples were collected from patients who had signed informed consent forms, which was approved by the Institutional Review Boards of Gangnam Severance Hospital. This study was additionally approved by the Office of Human Subjects Research at the National Institute of Health.

### Plasmid construction

For the generation of the pEGFP-human API5 (hAPI5) constructs, the DNA fragments encoding hAPI5 were amplified from cDNAs of CUMC6 tumor cells by PCR using a set of primers: 5′-GCAGATCTATGCCGACAGTAGAGGAGCT-3′ and 5′-GCGAATTCCTACTTCCCCTGAAGGTC-3′. The amplified DNAs were subsequently cloned into the *Bgl* II/*EcoR* I sites of pEGFP-C1 (Clontech, Mountain View, CA). Plasmid constructs were confirmed by DNA sequencing. The nucleotide sequences were determined using the BigDye Terminator Cycle Sequencing Ready Reaction Kit (Perkin Elmer Biosystems, Foster City, CA) and an ABI PRISM 377 DNA sequencer.

### Western blotting

A total of 5 × 10^5^ cells were used as described previously [18, 19]. Equal amounts of nuclear protein and cytosol protein were solubilized in Laemmli buffer (62.5 mM Tris/HCL pH 6.8, 10% glycerol, 2% SDS, 5% mercaptoethanol and 0.00625% bromophenol blue), boiled for 5 min, and then separated by 12% polyacrylamide gel electrophoresis and transferred to nitrocellulose membranes. The membranes were probed with primary antibodies of API5 (Sigma-Aldrich, St. Louis, MO; clone# 1C2, 1:250) or GAPDH (Chemicon International, Temecula, CA; clone no. 6C5, 1:5000) in Tris-buffered saline (TBS)-T containing 5% BSA (Sigma-Aldrich) at 4°C overnight, followed by 3 washes in TBST, 5 min per wash. The membranes were incubated with the appropriate secondary antibodies for 1 hr at room temperature. Immunoreactive bands were visualized by an enhanced chemiluminescence reaction (ECL, Elpis Biotech, Daejeon, Korea). To observe the cellular localization of API5, HeLa cells were subjected to fractionation using a commercial kit (Nuclear/Cytosol Fractionation Kit, Thermo scientific, Rockford, IL) according to the manufacturer's instructions.

### Immunofluorescence

In order to examine the cellular localization of API5, HeLa cells were cultured on 2-well Laboratory-Tek tissue culture chamber slides (BD Falcon, Bedford, MA) and transfected with 0.4 μg of pEGFP-hAPI5 using Lipofectamine™2000 (Invitrogen, Carlsbad, CA) according to manufacturer's protocol, and incubated for 24 hr. The transfected cells were fixed and permeable with Cytofix/Cytoperm (BD biosciences, San Diego, CA) for 20 min at 4°C. After washing in 1× Perm-wash buffer and counterstained nuclear with DAPI, localization of API5 was demonstrated under a confocal laser scanning microscope (ZEISS LSM700, Carl Zeiss, Oberkochen, Germany).

### Establishment of stable cell lines

CaSki, HeLa, and human embryonic kidney 293 (HEK 293) were obtained from the American Type Culture Collection (ATCC, Manassas, VA). To generate pcDNA3-API5 plasmid, DNA fragments encoding API5 were amplified from cDNAs of CUMC6 tumor cells by PCR and inserted into *Not* I site of pcDNA3 vector. Stable transfected lines were generated by transfecting no insert (pcDNA3) and pdDNA3-API5 vectors, selected and maintained in the presence of appropriate concentrations of Zeocin™ (Invitrogen).

### In vitro *transfection of siRNAs*

Synthetic small interfering RNA (siRNA) specific for Gfp or API5 was purchased from Invitrogen; Non-specific GFP, 5′-GCAUCAAGGUGAACUUCAA-3′ (sense), 5′-UUGA- AGUUCACCUUGAUGC-3′ (antisense); API5, 5′-UUACUGUGCUCUUAUAAGGAGG-3′ (sense), 5′-CCUCCUUCUUAUAAGAGCACAGUAA-3′ (antisense). HeLa cells (5 × 10^5^ cells/well on 6-well dish) were transfected with 300 pmol of the synthesized siRNAs using Lipofectamine 2000 (Invitrogen) according to the manufacturer’s instructions. RNAi was maintained 10–14 days after transfection of the siRNAs [18].

### Colony formation assay

The stable cell lines and siRNA transfected cells (500 cells/well) were plated onto 6 well tissue culture dishes and incubated for 2 weeks to allow colonies to develop. Media (4 ml/well) was replaced every 7 days. Colonies were stained with crystal violet (0.5% in methanol, Sigma-Aldrich) for 10 min, and washed with de-ionized water to remove excess stain. Stained colonies of diameter 1 mm were counted manually from microscopic images. Each colony formation assay was carried out in triplicate and repeated three times.

### Tissue microarray construction

TMAs were constructed from 479 formalin-fixed, paraffin-embedded tissue specimens, including 429 nonadjacent normal epithelial tissues. Some of the paraffin blocks were provided by the Korea Gynecologic Cancer Bank through Bio & Medical Technology Development Program of the Ministry of Education, Science and Technology, Korea. Briefly, hematoxylin and eosin (H&E) stained full-face sections of all cases were reviewed by an institutional pathologist to define representative tumor areas. Four 1.0 mm diameter tissue cores, consisting of matched tumor specimen and normal epithelial tissues, were retrieved from formalin-fixed, paraffin-embedded tissue blocks and arrayed on a 38 × 25 mm recipient paraffin block using a manual tissue arrayer MTA-1 (Beecher Instruments Inc., Silver Spring, MD). Sections were cut at 5 μm with a microtome and placed on charged glass slides. The presence of tumor tissues on the sections was verified by H&E staining.

### Immunohistochemical staining and scoring

The TMA sections were deparaffinized and rehydrated through xylenes and descending gradient alcohol. All slides were quenched for 10 min in 3% H_2_O_2_ to block for endogenous peroxidase. Heat-induced antigens retrieval was done for 10 min in an antigen retrieval buffer of pH 6 (Dako, Carpinteria, CA) using a steam pressure cooker (Pascal, Dako). Sections were then treated with protein blocks (Dako) for 20 min to block non-specific staining. The slides were then stained with anti-API5 mouse monoclonal antibody (Sigma-Aldrich, clone no. 1C2, 1:250 for 2 hr at room temperature) and rabbit anti-pERK1/2 monoclonal antibody (Cell Signaling, Danvers, MA; clone no. 20G11, 1:150 for 2 hr at room temperature) in a Dako Autostainer Plus (Dako). The antigen-antibody reaction was detected with Dako EnVision + Dual Link System-HRP (Dako) and DAB^+^ (3, 3′-Diaminobenzidine; Dako). After counterstaining in hematoxylin, slides were mounted manually and scanned using a ScanScope CS digital scanner (Aperio Technologies, Vista, CA).

The API5 and pERK1/2 staining results were scored based on (a) intensity [categorized as 0 (absent), 1 (weak), 2 (moderate), or 3 (strong)] and (b) the percentage of positively stained epithelial cells [scored as 0 (0-5% positive), 1 (6-25%), 2 (26-50%), 3 (51-75%), or 4 (>75%)]. A histoscore was generated by multiplying the mean intensity and percent scores (overall score range, 0–12). The histoscore was then dichotomized into low expression (histoscore, 0–6) and high expression (histoscore, 8–12). We selected a histoscores of 8 as the cutoff point for positive expression because histoscore of 8 or more matched with cases with strong or moderate intensity. Additional cut-points were not evaluated. For pERK1/2, nuclear and cytoplasmic staining was dichotomized into low expression (histoscore, 0–3) and high expression (histoscore, 4–12). Slides were scored without any clinical information, and the final staining score reported was the average of two independent pathologists.

### Statistical analysis

Statistical analyses of API5 and pERK1/2 expressions were performed using the Mann–Whitney test or the Kruskal-Wallis test. The *x*^2^-test was used to assess associations between molecular markers. Overall and disease-free survival curves were calculated according to the Kaplan-Meier method; survival analysis was performed using the log-rank test. The Cox proportional hazards model was used to estimate hazard ratios and confidence intervals in both univariate and multivariate models. Statistical analyses were done using SPSS version 18.0 (SPSS Inc., Chicago, IL). A value of *P* < 0.05 was considered statistically significant.

## Results

### Localization of API5 in cervical cancer cell lines

**Figure 1:**
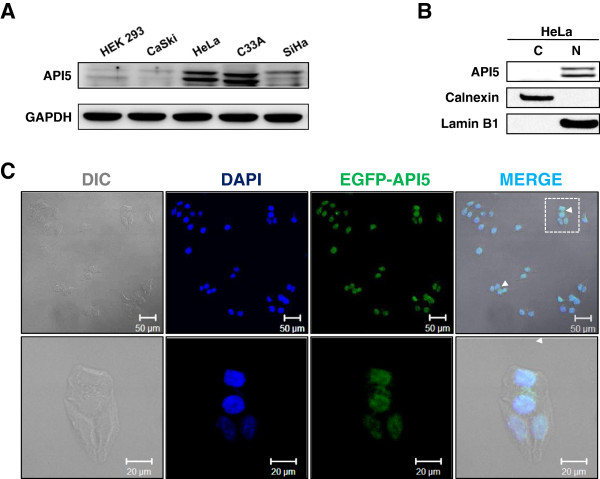
**API5 expression and its localization in various cervical cancer cell lines. (A)** Characterization of API5 expression in various cervical cancer cell lines by western blot analysis. **(B)** Nuclear and cytoplasmic fractions from HeLa cells were analyzed by western blot analysis. Calnexin and Lamin B1 were used as an index for cytosolic or nuclear fraction, respectively. **(C)** Confocal fluorescent microscopy was used to further evaluate the distribution of API5 in HeLa cells 24 hrs after transfection of pEGFP-API5. DAPI fluorescent dye was used for a nuclear counterstaining. Magnified images of boxed areas are shown in the lower panels. Arrowheads indicate cytoplasmic EGFP-API5 in the transfected HeLa cells.

The expression of human API5 was investigated in human cervical cancer cell lines using western blot analysis. HEK 293 human embryonic kidney epithelial cells were used as a control cell line representing non-tumorigenic cells. As shown in Figure 1A, API5 was detected as doublet bands, as has been reported in mammals [13]. Expression of API5 was most profound in HeLa and C33A while that in CaSki and SiHa was similar to non-turmorigenic HEK293 cells. We further analyzed the expression of API5 protein in cytoplasmic and nuclear fractions of the HeLa cells which have the highest expression of API5 among the cervical cancer cell lines examined by western blot analysis. As shown in Figure 1B, API5 was exclusively detected in the nuclear fraction. To further confirm the nuclear/cytosolic localization of API5, HeLa cells were transfected with pEGFP-Api5 DNA, and, in turn, examined with confocal laser scanning microscopy after counterstaining nuclear with DAPI. As shown in Figure 1C, we observed the dominant localization of API5 in nucleus although cytoplasmic API5 (indicated by arrowheads) was observed in small population of the transfected HeLa cells (less than 8%). We also observed a similar localization pattern of endogenous API5 in CaSki cells after immunofluorescence staining (Additional file 1: Figure S1). Taken together, these results demonstrate that API5 expresses in cervical cancer cell lines and is primarily localized in nucleus.

### The role of API5 in cell proliferation and colony formation in cervical cancer cell lines

**Figure 2:**
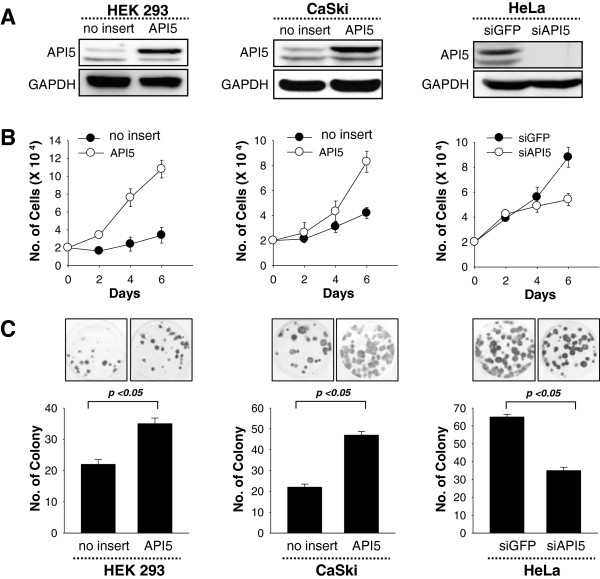
**API5 role in cell proliferation and colony formation of HEK293, CaSki, and HeLa cell lines. (A)** API5 protein expression was analyzed by western blot. **(B)** Proliferation assay: 2 × 10^4^ cells were plated in 24 well plates and cultured for additional 6 days. Cells were collected by trypsinization at the indicated times, and live cells were counted after trypan blue staining under a haemocytometer. **(C)** Colony formation: 500 cells were plated in 6 well plates and cultured for 2 weeks and formed colonies were stained with crystal violet. Data depicted as mean + s.e.m. from one representative experiment performed in triplicate.

To evaluate the effects of API5 on cell proliferation, the API5 expression vector or the control vector (no insert) were transfected into CaSki cells which have a low level of API5 expression. Non-tumorigenic HEK 293 cells with low background of API5 expression level were used as a positive control for comparison. Conversely, siRNA targeting API5 (siAPI5) or GFP (irrelevant negative control, siGFP) were also transfected into HeLa cells which have a high level of API5 expression. API5 expression level in the transfected cells were detected by western blotting (Figure 2A). The cell growth assay revealed that cell growth rate in both of the API5-transfected cells was significantly higher than control groups (Figure 2B). Similar increase was also observed in colony formation assay (Figure 2C). In contrast, knock-down of API5 in HeLa cells significantly decreased both of the cell growth rate and colony formation efficacy compared with siGFP control group (Figure 2B and C). These data demonstrate that API5 has a key role in cell proliferation and colony formation of cervical cancer cells.

### Clinicopathologic characteristics, API5 and pERK1/2 expression

To determine whether API5 overexpression is linked to clinical features of cervical cancer, we performed immunohistochemistry in a cohort of cervical tissues from patients with CIN or invasive cervical cancer. Patient ages ranged from 19 to 83 years (mean, 42.5 years). The clinicopathologic characteristics of the study are summarized in Additional file 2: Table S1. Tumor sizes ranged from 0.3 to 12.3 cm (mean 2.8 cm). The following histologic types were subjected: 141 squamous cell carcinomas (81.5%), 26 adenocarcinomas/adenosquamous carcinomas (15.0%), 5 small cell carcinomas (2.9%), and 1 clear cell carcinoma (0.6%). HC2-based HPV infection rate was 81.1% (55/67) in low grade CIN, 90.2% (157/174) in high grade CIN. The length of patient follow-up time ranged from 2 to 60 months, and median survival time at last follow-up was 40.5 months.

**Figure 3:**
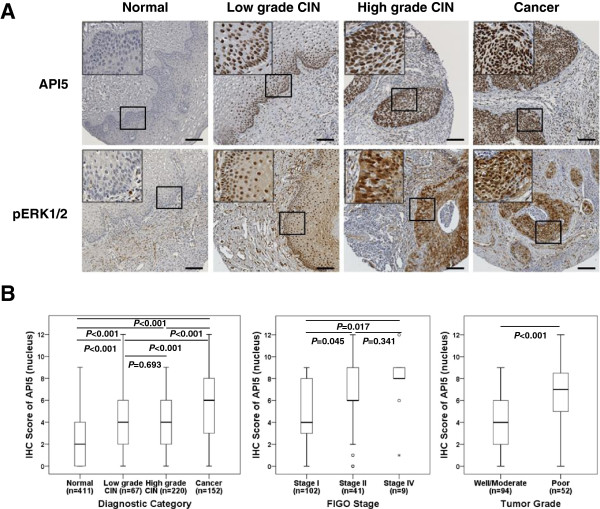
**API5 and pERK1/2 expression in human cervical neoplasias specimens. (A)** Representative immunohistochemical staining images of API5 and pERK1/2 in normal, low grade CIN, high grade CIN, and cervical cancer tissues. Bars: 100 μm. **(B)** API5 IHC staining score in cervical neoplasia samples. API5 IHC staining score in cervical cancer samples was significantly higher than that of all other groups. The mean API5 score associated directly with each tumor stage, stage I tumors stained more weakly than stage II and stage IV tumors. API5 IHC staining score in poorly differentiated cervical cancer samples was significantly higher than that of well/moderately differentiated cancers.

The TMA contains 173 cases of cervical cancer, however due to the complexity of sectioning, staining, as well as heterogeneity of the samples, only 152 and 150 samples could be interpreted for the API5 and pERK1/2, respectively. API5 protein expression was exclusively identified in the nucleus of tumor cells while the expression of pERK1/2 was detected in the cytoplasmic and nuclear of the tumor cells (Figure 3A). The expressions of API5 and pERK1/2 in relation to clinicopathologic characteristics were evaluated (Table 1). API5 expression gradually increased according to the phases of cervical cancer progression, from normal tissues through low and high grade CINs to cervical cancers (*P* < 0.001). Also, it correlated with features associated with advanced disease and poor outcome including FIGO stage (*P* = 0.004), tumor grade (*P* < 0.001), and chemo-radiation response (*P* = 0.004) (Figure 3B). In addition, subgroup analysis by stage was conducted for various stages of cervical cancer (Additional file 2: Table S2). The results were similar in all subgroups according to disease severity. The expression of pERK1/2 was up-regulated in high grade CIN and cancer specimens compared to normal and low grade CIN (*P* < 0.001, Table 1).

**Table 1:** **Expression of API5 and pERK1/2 in relation to clinicopathologic characteristics in IHC analysis**

			API5				pERK1/2	
	No.	%	Mean scores (95% CI)	***P value***	No.	%	Mean scores (95% CI)	***P value***
**All study subjects**	850	100	3.53 (3.35-3.71)		793	100	2.45 (2.27-2.63)	
**Diagnostic category**				<0.001				<0.001
Normal	411	48.3	2.44 (2.23-2.65)		382	48.1	1.69 (1.54-1.85)	
Low grade CIN	67	7.9	3.79 (3.23-4.36)		57	7.2	2.65 (2.17-3.13)	
High grade CIN	220	25.9	4.19 (3.88-4.50)		204	25.7	3.32 (2.95-3.69)	
Cancer	152	17.9	5.39 (4.92-5.87)		150	19.0	3.13 (2.53-3.73)	
**FIGO stage**				0.004				0.205
I	102	67.1	4.88 (4.35-5.42)		99	66.0	2.89 (2.16-3.62)	
II	41	27.0	6.17 (5.14-7.20)		44	29.3	3.30 (2.19-4.40)	
IV	9	5.9	7.67 (5.39-9.94)		7	4.7	5.43 (1.06-9.79)	
**Cell type**				0.533				0.114
SCC	122	80.3	5.32 (4.78-5.86)		124	82.7	3.35 (2.65-4.04)	
Others	30	19.7	5.70 (4.58-6.82)		26	17.3	2.08 (1.09-3.07)	
**Tumor grade**				<0.001				0.237
Well + Moderate	94	64.4	4.69 (4.12-5.26)		96	65.3	2.78 (2.07-3.49)	
Poor	52	35.6	6.62 (5.80-7.44)		51	34.7	3.53 (2.44-4.62)	
**Tumor size**				0.932				0.776
≤ 4 cm	105	69.1	5.38 (4.82-5.94)		103	68.7	3.07 (2.37-3.77)	
> 4 cm	47	30.9	5.43 (4.47-6.38)		47	31.3	3.26 (2.07-4.44)	
**LN metastasis**				0.385				0.637
No	104	76.5	5.00 (4.45-5.55)		102	75.0	2.71 (2.02-3.40)	
Yes	32	23.5	5.53 (4.24-6.82)		34	25.0	2.38 (1.25-3.52)	
**Chemoradiation**				0.004				0.216
Good response	29	80.6	3.41 (2.27-4.56)		31	81.6	1.39 (0.66-2.11)	
Bad response	7	19.4	7.00 (5.81-8.19)		7	18.4	0.43 (-0.07-0.92)	
**HPV test in CIN**				0.951				0.940
Negative	28	12.4	4.14 (3.19-5.10)		28	13.7	3.07 (2.12-4.02)	
Positive	198	87.6	4.17 (3.85-4.49)		177	86.3	3.11 (2.76-3.45)	

CIN, cervical intraepithelial neoplasia; FIGO, International Federation of Gynecology and Obstetrics; SCC, squamous cell carcinoma; LN, lymph node; HPV, human papillomavirus.

### Prognostic significance and association of API5 and pERK1/2 expression

Base on the fact that API5 is overexpressed, we examined the association between expression of API5 and pERK1/2 in cervical cancer or CIN specimens. Notably, expression of API5 was positively associated with the expression of pERK1/2 in both cancer (*P* < 0.001) and high grade CIN specimens (*P* = 0.031) (Table 2). However, there is no association between these two protein expressions in low grade CIN specimens. This data suggests that the expression of API5 and pERK1/2 is a closely coordinated event in incipient cervical cancer.

**Table 2:** **Association of API5 and pERK1/2 expression in cervical cancer and CIN patients**

	API5 expression					
	Low (-)	***%***	High (+)	***%***	Total no.	***P***value
**Cancer**	85	65.9	44	34.1	129	*P* < 0.001
pERK1/2 (-)	69	75.8	22	24.2	91	
pERK1/2 (+)	16	42.1	22	57.9	38	
**High grade CIN**	170	89.9	19	10.1	189	*P* = 0.031
pERK1/2 (-)	106	93.8	7	6.2	113	
pERK1/2 (+)	64	84.2	12	15.8	76	
**Low grade CIN**	51	96.2	2	3.8	53	*P* = 0.301
pERK1/2 (-)	33	94.3	2	5.7	35	
pERK1/2 (+)	18	100.0	0	0.0	66	

API5+, IHC score of ≥ 8; pERK1/2+, IHC score of ≥ 4.

**Figure 4:**
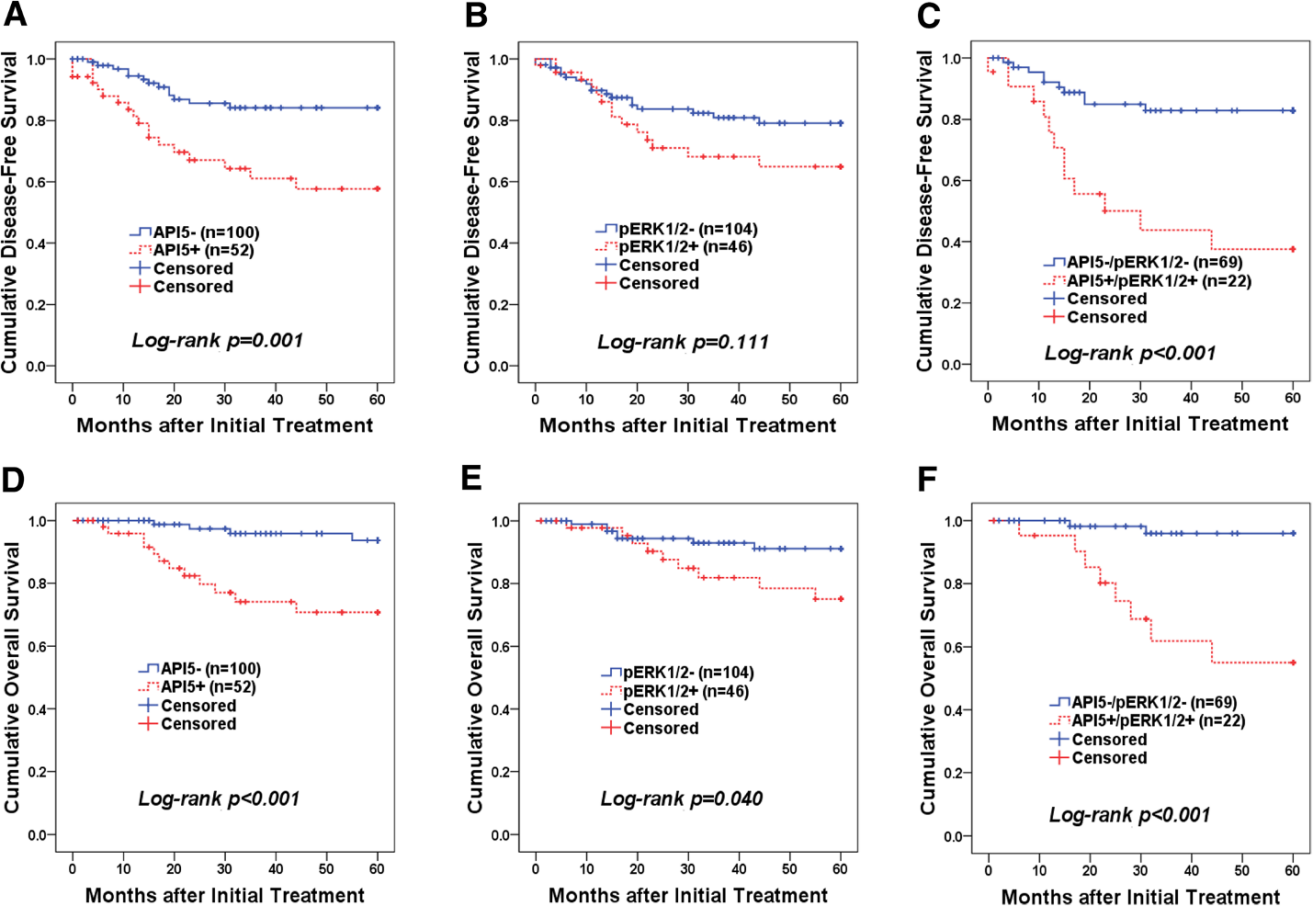
**Kaplan–Meier plots of disease-free survival (A - C) and overall survival (D - F) categorized based on API5 expression, pERK1/2 expression, and co-expression of API5/pERK1/2.** High API5 expression associated with short disease-free survival **(A**, ***P*** **= 0.001)** and overall survival rate **(D**, ***P*** **= 0.001).** Patients with high pERK1/2 expression displayed worse overall survival **(E**, ***P*** **= 0.040)**. The association of high API5/pERK1/2 with disease-free survival **(C)** and overall survival **(F)** was significantly different from that of low API5/pERK1/2 (*P* < 0.001 for both).

We next examined the relationship of API5 expression to patient outcome. As shown in Figure 4, Kaplan-Meier plots demonstrated that patients with high API5 expression (IHC score of ≥ 8) displayed significantly shorter disease-free survival (mean of 41.5 versus 53.0 months, *P* = 0.001) and overall survival (mean of 48.8 versus 58.4 months, *P <* 0.001) (Figure 4A and C). The high pERK1/2 expression (IHC score of ≥ 4) group had shorter survival whereas the low pERK1/2 expression group had longer survival in overall survival analysis (mean of 52.3 versus 56.7 months, *P* = 0.040) (Figure 4E). Furthermore, the patients with combined high API5 and high pERK1/2 expression showed significantly shorter disease-free survival (mean of 33.2 versus 52.2 months, *P <* 0.001) and overall survival (mean of 44.4 versus 58.6 months, *P <* 0.001) than patients who were low API5 and low pERK1/2 expression (Figure 4C and F). The independent prognostic significance of high API5 expression and a combined high API5 and high pERK1/2 expression as well as other clinicopathologic parameters was determined by applying Cox proportional hazards model. FIGO stage (*P* = 0.001), LN metastasis (*P* = 0.047), high API5 expression (*P* = 0.031), and a combination of high API5 and high pERK1/2 expression (*P* = 0.007) were related to shorter disease-free survival (Table 3). Furthermore, SCC cell type [hazard ratio = 0.23 (95% CI, 0.06-0.80), *P* = 0.021], LN metastasis [hazard ratio = 3.85 (95% CI, 1.08-13.73), *P* = 0.038], high API5 expression [hazard ratio = 3.98 (95% CI, 1.07-14.85), *P* = 0.039], and a combination of high API5 and high pERK1/2 expression [hazard ratio = 4.14 (95% CI, 1.12-15.21), *P* = 0.032] were the independent prognostic factors with respect to overall survival. Altogether, these data indicated that API5 expression serves as an important prognostic factor in human cervical cancer.

**Table 3:** **Univariate and multivariate analyses of the associations between prognostic variables and overall survival in 173 cases of cervical cancer**

Variables	Overall survival hazard ratio [95% CI], ***P***value		Disease-free survival hazard ratio [95% CI], ***P***value	
	Univariate analysis	Multivariate analysis	Univariate analysis	Multivariate analysis
**FIGO stage (≥IIb)**	3.10 [1.19-8.07], 0.020	1.62 [0.32-8.15], 0.557	5.74 [2.89-11.41], *<*0.001	5.86 [2.00-17.18], 0.001
**Cell type (SCC)**	0.30 [0.11-0.79], 0.015	0.23 [0.06-0.80], 0.021	0.84 [0.36-1.94], 0.695	NS
**Tumor grade (poor)**	2.75 [1.05-7.25], 0.040	1.80 [0.52-6.25], 0.353	2.09 [1.06-4.10], 0.031	1.05 [0.44-2.51], 0.901
**Tumor size (>4 cm)**	1.71 [0.65-4.51], 0.273	NS	2.54 [1.29-4.97], 0.007	1.28 [0.54-3.00], 0.568
**LN metastasis**	6.30 [2.10-18.87], 0.001	3.85 [1.08-13.73], 0.038	5.15 [2.38-11.13], <0.001	2.49 [1.02-6.17], 0.047
**API5+ (≥8)**	5.74 [1.82-18.05], 0.003	3.98 [1.07-14.85], 0.039	3.10 [1.52-6.34], 0.002	2.74 [1.09-6.85], 0.031
**pERK1/2+ (≥4)**	2.69 [1.00-7.23], 0.049	2.33 [0.65-8.32], 0.193	1.74 [0.86-3.51], 0.117	NS
**API5+/pERK1/2+**	5.02 [1.91-13.22], 0.001	4.14 [1.12-15.21], 0.032	4.56 [2.25-9.24], *<*0.001	3.99 [1.47-10.87], 0.007

FIGO, International Federation of Gynecology and Obstetrics; SCC, squamous cell carcinoma; LN, lymph node; NS, not significant.

## Discussion

API5 was originally identified as an apoptosis inhibitory protein whose expression prevents apoptosis after growth factor deprivation [10]. Recently, few studies have addressed the API5 mechanism other than its anti-apoptotic effects [14, 15, 20]. The putative oncogenic role of API5 was suggested by Kim and colleagues in cervical cancer cell lines [20]. They reported that API5 overexpression could promote cell growth and invasiveness of cervical cancer cell line CUMC-6 [20]. However they did not clearly rule out the effect of endogenous API5 expression without the application of API5 gene knockdown or silencing. In this study, API5 protein levels were examined by fractionated immunoblotting in HeLa and CaSki cervical cell lines. API5 protein was predominantly expressed in nuclear fraction of HeLa compared to CaSki, even without apoptotic stimuli (Figure 1). To investigate the role of API5 on cell growth and clonogenicity, API5 protein was overexpressed using a wild type API5 expression vector in CaSki as well as in non-tumorigenic HEK 293 cells. Overexpressed API5 mediated an increase of cell proliferation in CaSki cell line. On the other hand, inhibition of API5 expression by API5 siRNA gene knockdown resulted in significant inhibition of cell growth in HeLa cells. These results demonstrated that API5 overexpression is closely linked to cancer cell proliferation, suggesting that API5 cloud contributes to the development of cervical cancer.

Epidermal Growth Factor (EGF), Insulin Growth Factor 1 (IGF-1) and Vascular Endothelial Growth Factor (VEGF) are known to regulate cervical cancer cell proliferation and invasiveness which play key roles in determining the high-risk factors and lead to recurrence and mortality [21]. The expression of growth factor receptor is also increased in cervical cancer tissues and cancer cells. EGF Receptor (EGFR) is related to HPV infection as EGFR cytoplasmic expression increases with increasing grade of CIN [22]. IGF-1 receptor (IGF-1R) expression level is elevated in cervical cancer cell cultures [23]. However, the clinical utility of EGFR expression as a biomarker for prognosis or for treatment of cervical cancer is not defined, as normal cervical epithelium also expresses EGFR at various levels [24], and this expression is not correlated with the HPV type [22]. The correlation between IGF-1R expression and cervical cancer stages is also limited in early-stage cervical cancer [25]. Thus, several studies have been focusing on signaling pathway, which can be activated by growth factors. Activation of ERK/MAPK cascade by growth factors has been reported in many human carcinomas, including cervical cancer cell lines [26–29]. One critical down-stream molecule is phosphorylated ERK1/2 [30–32]. Previous studies suggested a relationship between API5 and growth factors, such as Fibroblast Growth Factor (FGF) [13, 14], which can activate ERK/MAPK signaling cascade. Furthermore, we recently demonstrated that API5 activates ERK through FGF/FGFR1 pathway [16]. In agreement with this study, we observed that API5 expression levels were positively associated with ERK1/2 phosphorylation both in high grade CIN and cancer specimens. In addition, API5 expression positively correlated with disease severity in cervical neoplasias and negatively with overall survival of cervical cancer patients. Taken together, our data suggest that API5 may play an important role in the progression of cervical neoplasia through ERK/MAPK activation.

As an anti-apoptotic protein, API5 may be closely implicated in the regulation of apoptosis and is highlighted as a potential drug target in cancer. In this context, API5 expression has been reported in a number of tumor cell lines [20], and, subsequently, API5 elevated mRNA and protein expression levels were confirmed in a wide variety of transformed cell lines [11, 13, 20, 33, 34]. Sasaki and colleagues reported that an increased *API5* mRNA expression was detected in 12.7% (12/94) of the non-small cell lung cancer (NSCLC) biopsies and its presence was correlated with poor survival, especially in patients with squamous cell lung cancer [15]. Likewise, Wang et al. investigated the prognosis characteristics of API5 and used real-time RT-PCR to characterize *API5*, casein kinase 2 α subunit (*CSNK2A1*), and *NME1* transcripts in 145 NSCLC cases [35]. They showed that a combination of high *CSNK2A1* and high *API5* mRNA expressions was predictive of poor prognosis in NSCLC patients [35]. Kim et al. previously reported *API5* gene up-regulation in some metastatic lesions in lymph node tissues and did not find API5 overexpression in primary cervical cancer tissues, due to the small number of examined cases [20]. In spite of supporting evidence of *API5* gene involvement in tumorigenesis, the information about its protein expression in human tumors is still scarce. Recently, Koci and colleagues analyzed API5 protein expression in a variety of human carcinomas by western blotting [36]. They detected API5 protein expression in biopsies of lung (23%, 3/13) and colorectal tumors (33%, 9/27). In the current study, we observed increased protein expression of API5 in 34.2% (52/152 cases) of cervical cancers, and API5 expression level gradually increased during the transition from normal tissue to cervical carcinoma (Table 1), suggesting an important role of API5 in cervical tumor progression.

Regarding cancer cell differentiation, we observed that higher API5 protein expression in poorly differentiated carcinomas (mean score = 6.62) than in well and moderately differentiated carcinomas (mean score = 4.69). It is reasonable to deduce that up-regulated API5 expression was more frequent in poorly differentiated carcinomas as API5 putative invasiveness has been reported in cervical cancer cell lines [20]. In addition, API5 expression positively correlated with cervical cancers resistance to chemo-radiation therapy. These data underscore the value of API5 expression as a high risk factor in cervical cancer. Clinically, concurrent platinum-based chemo-radiation has become the mainstay for treating locally advanced cervical cancers. Although chemo-radiation has significantly improved both disease-free and overall survival of cervical cancer patients when compared to radiation alone, the 5-year overall survival of patients treated primarily with combination therapy is still under 70% [2]. Currently, no known molecular marker can accurately predict the response to platinum-based chemo-radiation or outcome in cervical cancer. However, we showed that API5 expression in cervical cancer specimens positively correlated with resistance to chemo-radiation therapy, implicating API5 overexpression as a strong risk factor for poor outcomes in cervical cancer. Thus, our study suggests the possibility that the effect of chemo-radiation can be compromised in cervical cancer patients with API5 overexpression, an observation that can have profound clinical implications. Further research regarding the molecular mechanisms responsible for API5-associated resistance to chemo-radiation will hopefully provide new insights, allowing the identification of new potential targets for therapy and the design of better treatment strategies. Although there was a small increase in API5 expression in LN metastasis cases, when compared with those without, we did not find a correlation between API5 expression and the presence of LN metastasis. This may be due to the smaller sample size of LN metastasis cases (*n* = 37) when compared to that of no metastasis (*n* = 119). We also included HPV infection in the analysis and observed no statistical difference in API5 expression between HPV-positive (*n* = 198) and -negative (*n* = 28) patients. Therefore, further studies are needed to establish the relationship between API5 and the aggressiveness of cervical cancer cells, and to identify the exact causation of API5 overexpression in cervical cancer.

Our study also showed that API5 expression in cervical cancer was associated with poor overall and disease-free survival. Patients with high levels of API5 expression had an increased risk of disease progression and death. In addition, API5 expression was an independent prognostic factor for overall and disease-free survival after adjusting for well-known prognostic parameters including FIGO stage, cell type, tumor grade, tumor size and LN metastasis. In agreement with our study, poor prognosis has been reported in a study of 94 NSCLC patients with increased API5 expression [15]. To the best of our knowledge, this is the first study designed to evaluate the association between API5 expression and clinicopathologic variables, including survival of cervical cancer patients. Our results not only suggest the promising potential of API5 as a prognostic and survival marker, but also warrant further studies on a possible link between the biological function of API5 and the pathogenesis of cervical cancer.

## Conclusions

In conclusion, we demonstrated that API5 plays a key role in cell proliferation and colony formation. Subsequently, we observed that API5 protein is overexpressed in human cervical cancer tissue specimens. API5 protein expression levels were found to significantly correlate with the prognosis of cervical cancer, as high level of API5 protein expression in cervical cancer lesions is closely associated with advanced tumor stage and grade, and shorter overall survival for the patients. Overall, our study suggests that overexpression of API5 is a common features in cervical cancer and might represent a novel prognostic marker for the disease.

## Electronic supplementary material

Additional file 1: Figure S1: Localization of endogenous API5 in CaSki cells. Confocal fluorescent microscopy was used to evaluate the distribution of endogenous API5 in CaSki cells. The cells were fixed, permeabilized, and then immunostained with anti-API5 antibody (Santa Cruz, USA; H-300, 1: 250) at 4°C for overnight. After washing with PBS, the cells were further incubated with Alexa Flour 488-conjugated goat anti-rabbit IgG (Invitrogen) for 1 hr at room temperature, followed by washing with PBS, and then analyzed using a Confocal fluorescent microscopy. DAPI fluorescent dye was used for a nuclear counterstaining. (PDF 23 KB)

Additional file 2: Table S1: Clinicopathologic characteristics of cases. **Table S2.** API5 expression in various stages of cervical cancer. (DOC 50 KB)

## Abbreviations

API5: apoptosis inhibitor-5
HPV: Human papillomavirus
ERK1/2: extracellular-signal-regulated kinases 1/2
MAPK: Mitogen activated protein kinase
AAC11: Anti-apoptosis clone 11
FIF: Fibroblast growth factor-2-interacting factor
CIN: Cervical intraepithelial neoplasia
TMA: Tissue microarray
H&E: Hematoxylin and eosin
EGF: Epidermal growth factor
IGF-1: Insulin growth factor 1
VEGF: Vascular endothelial growth factor
FGF: Fibroblast growth factor
NSCLC: Non-small cell lung cancer
LN: Lymph node
SCC: Squamous cell carcinoma.
