# Supplementary figures and images for: Apoptosis inhibitor-5 overexpression is associated with tumor progression and poor prognosis in patients with cervical cancer
Source: BMC Cancer. 2014 Jul 28;14:545. doi: 10.1186/1471-2407-14-545 (PMC4125689; doi:10.1186/1471-2407-14-545)

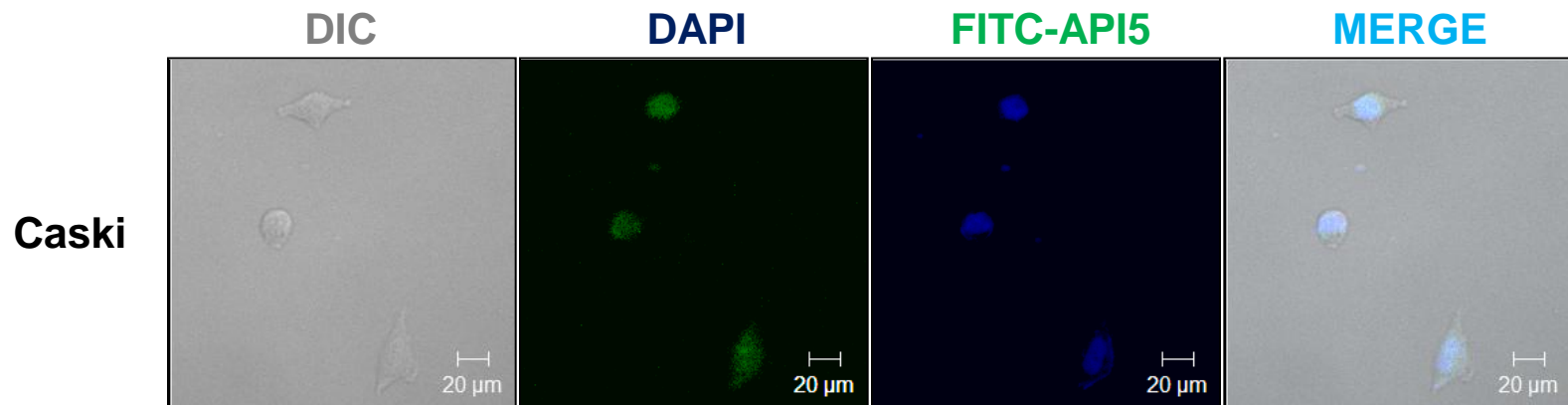

Supplement: Supplementary file 1 — Additional file 1: Figure S1: Localization of endogenous API5 in CaSki cells. Confocal fluorescent microscopy was used to evaluate the distribution of endogenous API5 in CaSki cells. The cells were fixed, permeabilized, and then immunostained with anti-API5 antibody (Santa Cruz, USA; H-300, 1: 250) at 4°C for overnight. After washing with PBS, the cells were further incubated with Alexa Flour 488-conjugated goat anti-rabbit IgG (Invitrogen) for 1 hr at room temperature, followed by washing with PBS, and then analyzed using a Confocal fluorescent microscopy. DAPI fluorescent dye was used for a nuclear counterstaining. (PDF 23 KB) [file 12885_2014_4736_MOESM1_ESM.pdf]
