# Supplementary material for: Apoptosis inhibitor-5 overexpression is associated with tumor progression and poor prognosis in patients with cervical cancer
Source: BMC Cancer. 2014 Jul 28;14:545. doi: 10.1186/1471-2407-14-545 (PMC4125689; doi:10.1186/1471-2407-14-545)
Supplement: Supplementary file 2 — Additional file 2: Table S1: Clinicopathologic characteristics of cases. Table S2. API5 expression in various stages of cervical cancer. (DOC 50 KB) [file 12885_2014_4736_MOESM2_ESM.doc]

**Supplementary Table 1 Clinicopathologic characteristics of the cases**

| **Category** | **No.**  **of cases** | **Percentage of total** |
| --- | --- | --- |
| **Diagnostic category** |  |  |
| Normal | 429 | 47.2 |
| Low grade CIN | 71 | 7.8 |
| High grade CIN | 235 | 25.9 |
| Cancer | 173 | 19.1 |
| **FIGO stage** |  |  |
| I | 116 | 67.1 |
| II | 48 | 27.7 |
| IV | 9 | 5.2 |
| **Tumor type** |  |  |
| SCC | 141 | 81.5 |
| Adenocarcinoma | 19 | 10.9 |
| Adenosquamous | 7 | 4.1 |
| Small cell | 5 | 2.9 |
| Clear cell | 1 | 0.6 |
| **Tumor grade** |  |  |
| Well | 3 | 1.8 |
| Moderate | 106 | 63.9 |
| Poor | 57 | 34.3 |
| **LN metastasis** |  |  |
| No | 119 | 76.3 |
| Yes | 37 | 23.7 |
| **Chemoradiation response** |  |  |
| Good | 35 | 79.5 |
| Bad | 9 | 20.5 |
| **HPV test in CIN** |  |  |
| Negative | 29 | 12.0 |
| Positive | 212 | 88.0 |

CIN, cervical intraepithelial neoplasia; FIGO, International Federation of Gynecology and Obstetrics; SCC, squamous cell carcinoma; LN, lymph node; HPV, human papillomavirus

**Supplemental Table 2 API5 expression in various stages of cervical cancer**

|  | **No. of cases** | **% of total** | **API5 expression** | |
| --- | --- | --- | --- | --- |
|  |  |  | **Low (-)** | **High (+)** |
| **Stage IA** | 14 | 9.2 | 11 (78.6%) | 3 (21.4%) |
| **Stage IA+ IB** | 102 | 67.1 | 73 (71.6%) | 29 (28.4%) |
| **Stage IA+ IB+IIA** | 110 | 72.4 | 76 (69.1%) | 34 (30.9%) |
| **Stage IA+ IB+IIA+IIB** | 143 | 94.1 | 98 (68.5%) | 45 (31.5%) |
| **Stage IA+ IB+IIA+IIB+IVA** | 148 | 97.4 | 100 (67.6%) | 48 (32.4%) |
| **Stage IA+ IB+IIA+IIB+IVA+IVB** | 152 | 100 | 100 (65.8%) | 52 (34.2%) |

API5+, histoscore of ≥ 8
